# Comprehensive genotoxicity and carcinogenicity assessment of molnupiravir

**DOI:** 10.1093/toxsci/kfae112

**Published:** 2024-09-20

**Authors:** Patricia A Escobar, Zhanna Sobol, Randy R Miller, Sandrine Ferry-Martin, Angela Stermer, Binod Jacob, Nagaraja Muniappa, Rosa I Sanchez, Kerry T Blanchard, Alema Galijatovic-Idrizbegovic, Rupesh P Amin, Sean P Troth

**Affiliations:** Nonclinical Drug Safety and Pharmacokinetics Dynamics Metabolism and Bioanalysis, Preclinical Development, Merck & Co. Inc., Rahway, NJ 07065, United States; Nonclinical Drug Safety and Pharmacokinetics Dynamics Metabolism and Bioanalysis, Preclinical Development, Merck & Co. Inc., Rahway, NJ 07065, United States; Nonclinical Drug Safety and Pharmacokinetics Dynamics Metabolism and Bioanalysis, Preclinical Development, Merck & Co. Inc., Rahway, NJ 07065, United States; Nonclinical Drug Safety and Pharmacokinetics Dynamics Metabolism and Bioanalysis, Preclinical Development, Merck & Co. Inc., Rahway, NJ 07065, United States; Nonclinical Drug Safety and Pharmacokinetics Dynamics Metabolism and Bioanalysis, Preclinical Development, Merck & Co. Inc., Rahway, NJ 07065, United States; Nonclinical Drug Safety and Pharmacokinetics Dynamics Metabolism and Bioanalysis, Preclinical Development, Merck & Co. Inc., Rahway, NJ 07065, United States; Nonclinical Drug Safety and Pharmacokinetics Dynamics Metabolism and Bioanalysis, Preclinical Development, Merck & Co. Inc., Rahway, NJ 07065, United States; Nonclinical Drug Safety and Pharmacokinetics Dynamics Metabolism and Bioanalysis, Preclinical Development, Merck & Co. Inc., Rahway, NJ 07065, United States; Nonclinical Drug Safety and Pharmacokinetics Dynamics Metabolism and Bioanalysis, Preclinical Development, Merck & Co. Inc., Rahway, NJ 07065, United States; Nonclinical Drug Safety and Pharmacokinetics Dynamics Metabolism and Bioanalysis, Preclinical Development, Merck & Co. Inc., Rahway, NJ 07065, United States; Nonclinical Drug Safety and Pharmacokinetics Dynamics Metabolism and Bioanalysis, Preclinical Development, Merck & Co. Inc., Rahway, NJ 07065, United States; Nonclinical Drug Safety and Pharmacokinetics Dynamics Metabolism and Bioanalysis, Preclinical Development, Merck & Co. Inc., Rahway, NJ 07065, United States

**Keywords:** mutagenicity, genotoxicity, antiviral, β-D-N4-hydroxycytidine (NHC), carcinogenicity, molnupiravir

## Abstract

Molnupiravir is registered or authorized in several countries as a 5-d oral coronavirus disease 2019 treatment for adults. Molnupiravir is a prodrug of the antiviral ribonucleoside β-D-N4-hydroxycytidine (NHC) that distributes into cells, where it is phosphorylated to its pharmacologically active ribonucleoside triphosphate (NHC-TP) form. NHC-TP incorporates into severe acute respiratory syndrome coronavirus 2 RNA by the viral RNA-dependent RNA polymerase, resulting in an accumulation of errors in the viral genome, leading to inhibition of viral replication and loss of infectivity. The potential of molnupiravir to induce genomic mutations and DNA damage was comprehensively assessed in several *in vitro* and *in vivo* genotoxicity assays and a carcinogenicity study, in accordance with international guideline recommendations and expert opinion. Molnupiravir and NHC induced mutations *in vitro* in bacteria and mammalian cells but did not induce chromosome damage in *in vitro* or *in vivo* assays. The *in vivo* mutagenic and carcinogenic potential of molnupiravir was tested in a series of *in vivo* mutagenicity studies in somatic and germ cells (Pig-a Assay and Big Blue^®^ TGR Mutation Assay) and in a carcinogenicity study (transgenic rasH2-Tg mouse), using durations of exposure and doses exceeding those used in clinical therapy. *In vitro* genotoxicity results are superseded by robustly conducted *in vivo* studies. Molnupiravir did not increase mutations in somatic or germ cells in the *in vivo* animal studies and was negative in the carcinogenicity study. The interpretation criteria for each study followed established regulatory guidelines. Taken together, these data indicate that molnupiravir use does not present a genotoxicity or carcinogenicity risk for patients.

Molnupiravir is an orally administered, small-molecule ribonucleoside prodrug with *in vitro* and *in vivo* activity against a range of RNA viruses and a high barrier to the development of antiviral resistance (Strizki et al. 2023). Molnupiravir is authorized for use under Emergency Use Authorization, conditional or provisional marketing authorization, or special approval by regulatory authorities in various countries worldwide. The efficacy of molnupiravir (800 mg every 12 h for 5 d) was demonstrated in the pivotal phase 3 component of the MOVe-OUT trial, in which nonhospitalized, unvaccinated adult patients with mild-to-moderate coronavirus disease 2019 (COVID-19) at high risk of progression to severe COVID-19 (including hospitalization or death) were enrolled (Jayk Bernal et al. 2022). The ability of molnupiravir to reduce the incidence of patient hospitalization or death through day 29 was demonstrated clinically (Jayk Bernal et al. 2022). Real-world evidence studies have shown the effectiveness of molnupiravir across a broad range of patient populations with mild-to-moderate COVID-19, from highly vaccinated individuals at lower risk of poor outcomes to clinically vulnerable individuals at the highest risk of poor outcomes, including death. These real-world clinical data, collected largely when Omicron was the predominant variant of severe acute respiratory syndrome coronavirus 2 (SARS-CoV-2), provide compelling evidence of the effectiveness of molnupiravir for treatment of patients across a continuum of risk (Wong et al. 2022; Bajema et al. 2023; Van Heer et al. 2023; Wai et al. 2023; Xie et al. 2023).

Molnupiravir is rapidly absorbed and extensively hydrolyzed to the ribonucleoside analog β-D-N4-hydroxycytidine (NHC) before reaching systemic circulation. NHC is a substrate of nucleoside uptake transporters, which results in its rapid and broad distribution into cells following conversion to its pharmacologically active triphosphate form (NHC-TP) by host kinases (Fig. 1). As a substrate of the viral RNA-dependent RNA polymerase, NHC-TP is incorporated into the viral genome, increasing proofreading error rates during viral RNA replication. After sufficient accumulation of viral RNA mutations has been reached—viral error induction—only nonviable virus is produced, resulting in an overall reduction in the infective virus population (Kabinger et al. 2021). Although the ribonucleotide analog, NHC, is not expected to incorporate into genomic DNA given the robust and conserved mechanisms that exclude ribonucleotides from DNA (Williams et al. 2016), the incubation of cells with NHC *in vitro* induces mutations in bacterial (Salganik et al. 1973; Janion and Glickman 1980; Negishi et al. 1988), fungal (de Serres and Brockman 1993), and mammalian cells (Zhou et al. 2021; Miranda et al. 2022). However, *in vitro* assays often do not accurately replicate *in vivo* cellular activities, including metabolism, regulation, and replication processes, and do not account for extracellular factors, such as absorption, distribution, and excretion, all of which are comprehensively represented using *in vivo* systems. This is particularly relevant for NHC, which is rapidly eliminated *in vivo*, primarily by metabolism to native nucleosides (cytidine [C] and uridine [U]) (Hernandez-Santiago et al. 2004) (Fig. 1). Consistent with these considerations, *in vitro* genotoxicity results are superseded by robustly conducted *in vivo* studies. According to regulatory guidelines and expert opinion, *in vivo* mutation assays (such as the *in vivo* rodent transgenic mutation assay) are recommended as follow-up to assess mutagenic risk when a compound induces mutations *in vitro* in bacteria or mammalian cells (Eastmond et al. 2009; International Council for Harmonisation (ICH) Harmonised Tripartite Guideline 2011; Robison et al. 2021). Negative results in appropriate *in vivo* mutation assays and demonstration of exposure are considered sufficient to conclude that there is an absence of significant mutagenicity risk (International Council for Harmonisation (ICH) Harmonised Tripartite Guideline 2011). Furthermore, the conduct of the carcinogenicity study with molnupiravir is expected to resolve any concerns relating the carcinogenic risk empirically associated with Ames-positive compounds.

**Fig. 1.**
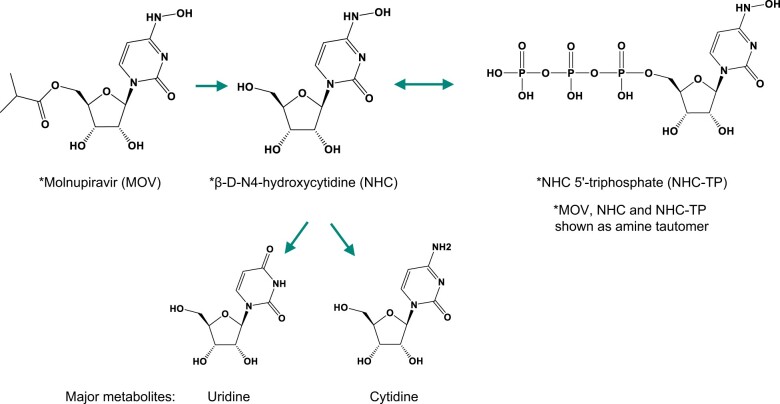
Major routes of molnupiravir metabolism.

Here, we describe a comprehensive genotoxicity and carcinogenicity assessment of molnupiravir.

## Materials and methods

### In vitro studies

All studies were conducted using good laboratory practices (GLPs) and in accordance with relevant International Council for Harmonization of Technical Requirements for Pharmaceuticals for Human Use (ICH) (ICH S1R1) and Organization for Economic Co-operation and Development (OECD) test guidelines (TG; OECD TG 471: Bacterial Reverse Mutation Test and OECD TG 487: In Vitro Mammalian Cell Micronucleus Test), with the exception of the bacterial reverse mutation (Ames) test with NHC, which was non-GLP.

### Bacterial reverse mutation (Ames) test

Molnupiravir (EIDD-2801, lot no. R00002-87-35; lot no. 60401-19-001; lot no. 000A010, stored refrigerated [2°C to 8°C]) and NHC (EIDD-1931, lot no. MAL013-291, stored refrigerated [2°C to 8°C]) were tested using a plate-incorporation procedure for *in vitro* bacterial reverse mutation assays in *Salmonella typhimurium* strains (TA1537, TA98, TA100, and TA1535) and an *Escherichia coli* strain (WP2 *uvr*A). Molnupiravir was also tested in *Salmonella typhimurium* strain TA102. Molnupiravir and NHC were tested up to 5,000 μg/plate with and without metabolic activation (Aroclor 1254-induced rat liver S9) (Molecular Toxicology, Inc., Boone, NC). The same plate-incorporation procedure was followed for molnupiravir and NHC; however, with molnupiravir the procedure was performed in triplicate and under GLP, and for NHC the testing was performed in duplicate and under non-GLP. This test was considered positive for mutagenicity when the test article treatment induced an increase in the number of revertants per plate with an increasing concentration of at least 2 times the vehicle control background frequency for strains with high spontaneous levels (TA100 and TA102) and 3 times for those with low spontaneous levels (TA1537, TA98, TA1535, and WP2 *uvr*A).

### In vitro micronucleus assay

Molnupiravir was tested in an *in vitro* micronucleus assay in a human lymphoblastoid cell line, TK6 cells, with and without metabolic activation (Aroclor 1254-induced rat liver S9) at concentrations that ranged from 0.650 to 330 μg/ml (∼1 mM); 1 mM (330 μg/ml) is the highest concentration recommended by ICH guidance. The cells were originally obtained from the American Type Culture Collection (ATCC, Rockville, MD) and transferred to the testing laboratory. The description of the cells and validation of this OECD TG 487-compliant method has been previously described (Sobol et al. 2012). TK6 stock cultures were maintained in culture media, Roswell Park Memorial Institute 1640 + L-glutamine, supplemented with heat-inactivated fetal bovine serum, 10%, and penicillin-streptomycin, 1%. TK6 cells were set up in vented T-75-cm^2^ flasks at a cell density of 1.3 to 1.8 × 10^5^ cells/ml.

TK-6 cells were treated with molnupiravir, vehicle, or a positive control with or without metabolic activation for short incubation periods (4 h) and without activation for long incubation periods (27 h). Positive controls were mitomycin C (CAS no. 50-07-7; Sigma-Aldrich, Inc., St Louis, MO, lot no. SLBX1864) at 0.125 and 0.0625 μg/ml in 4-h cultures without metabolic activation, vinblastine (CAS no. 143-67-9; Sigma-Aldrich, Inc., lot no. 028M4095V and lot no. 107M4057V) at 0.003 and 0.0025 μg/ml in 27-h cultures without metabolic activation, and cyclophosphamide (CAS no. 6055-19-2; Sigma-Aldrich, Inc., lot no. MKCG5464) at 11.9 and 4.7 μg/ml in 4-h cultures with metabolic activation. A harvest time of approximately 27 h was used for the 27-h exposure without metabolic activation. A harvest time of approximately 44 h was used for the 4-h exposure with and without metabolic activation, with a 40-h recovery period.

Acridine orange-stained slides were prepared for each culture selected for microscopic micronucleus evaluation after hypotonic treatment and fixation. Slides were blind-coded for scoring. Micronucleus frequencies were analyzed in 1,000 cells per culture (2,000 cells per concentration). Cytotoxicity was determined based on relative population doubling compared with the concurrent vehicle control as described in OECD TG 487.

The micronucleus assay result is considered positive for inducing micronuclei when a statistically significant increase (*P* *≤* 0.05) in the mean percentage of micronucleated cells is observed at 1 or more test article dose levels, a dose-response is observed (statistically significant for dose-response trend on the Cochran–Armitage test if *P* *≤* 0.05), and the increase is outside the historical control range of the vehicle control. Statistical analysis was performed using a one-tailed Fisher’s exact test on the total number of micronucleated cells, comparing the treated groups to the results obtained from the concurrent vehicle control group as previously described (San et al. 2002).

### In vivo studies

Animal studies were conducted under GLP and in accordance with relevant ICH (ICH S2R1 and ICH S1) and OECD guidelines (TG 474 and TG 488). Procedures were performed in facilities using procedures approved by the respective laboratory institutional animal care and use committees.

### Rat bone marrow micronucleus assay

Molnupiravir 0, 500, 1,000, and 2,000 mg/kg/d once daily by oral gavage was administered for 2 consecutive days to 5 Sprague–Dawley Crl: CD(SD) male and female rats. Molnupiravir and vehicle control (methylcellulose, 1% [weight/volume (w/v)], in deionized water) formulations were administered at a dose volume of 10 ml/kg. The highest dose tested, 2,000 mg/kg, is the dose limit per the ICH S2R1 and the OECD Test Guideline 474. The positive control (cyclophosphamide monohydrate) was administered once.

Approximately 24 h after the last molnupiravir dose, all animals were humanely euthanized by inhalation of carbon dioxide. Bone marrow was collected by aspiration or flushing using a syringe that contained heat-inactivated fetal bovine serum. The bone marrow was centrifuged, and resuspended pellets were used to prepare slides. Acridine orange-stained slides were prepared for each animal that was selected for microscopic micronucleus evaluation. Slides were blind-coded for scoring.

Two evaluations were made for each slide: (i) the polychromatic erythrocyte (PCE)/total erythrocyte ratio was determined in 500 erythrocytes scored per animal, and (ii) the percentage of micronucleated PCEs was determined in 4,000 PCEs scored per animal. Control and treated groups were compared using the one-way analysis of variance (ANOVA) *F* test with the Dunnett correction. An additional one-way ANOVA test was conducted on log-transformed MN frequency data, and the results of the statistical analysis were consistent between the two ANOVA tests. The Cochran–Armitage test was used for the determination of a dose-response trend. In addition, the positive and vehicle control groups were compared using a separate parametric one-way ANOVA. A result was considered significant if *P* ≤ 0.05. The result of the test was considered clearly positive when at least 1 treatment group exhibited a statistically significant increase in the percentage of micronucleated PCEs compared with the concurrent negative control (*P* *≤* 0.05), the increase was dose-related (*P* *≤* 0.05), and any increase was outside the 95% control interval of the historical negative control data. The assay was considered clearly negative when none of these criteria were met. The result was considered equivocal when neither the positive nor negative criteria were met.

### In vivo erythrocyte Pig-a gene mutation assay

The *in vivo* peripheral blood erythrocyte Pig-a mutation assay was conducted after administration of molnupiravir 0, 50, 150, and 500 mg/kg/d once daily or vehicle control (methylcellulose, 1% [w/v], in deionized water) by oral gavage for 28 consecutive days at a dose volume of 10 ml/kg to at least 5 Sprague–Dawley Crl: CD(SD) male rats. The high dose of 500 mg/kg/d was the maximum tolerated dose.

On day 29 after treatment initiation, animals were humanely euthanized by inhalation of carbon dioxide. Blood samples were collected through the abdominal aorta and subsequently processed, stained, and analyzed. Based on the procedures described in the MutaFlow (Litron Laboratories, Rochester, NY) *Pig-a* mutation analysis instruction manual of the rat blood kit (MutaFlowPLUS; rat, 96-well based) and described by Dertinger et al. (2011). Immature erythrocytes known as reticulocytes (RETs) or erythrocytes (RBCs), derived from rapidly proliferating erythroid precursor cells of the bone marrow were analyzed using flow cytometry for the Pig-a mutant phenotype (loss of cell surface marker) by labeling with fluorochrome-conjugated antibodies against glycosylphosphatidylinositol (GPI) proteins. Although Pig-a wild-type cells fluoresce, Pig-a mutant cells are nonfluorescent because of a loss of the GPI-anchored epitope (Gollapudi et al. 2015).

The Levene’s test was used to assess the homogeneity of group variances. Control and treated groups were compared using the one-way ANOVA *F* test with the Dunnett’s correction, and pairwise analysis was used to compare the vehicle control and positive control groups. A result was considered significant if *P* *≤* 0.05.

The Pig-a Assay was considered clearly positive when all the following criteria were met: (i) at least 1 of the test article groups exhibited a statistically significant (*P* ≤ 0.05) increase in the frequency of mutant RETs or mutant RBCs compared with the concurrent vehicle control and (ii) exhibited a dose-related increase when evaluated with an appropriate trend test, and (iii) if any of the results were outside the distribution of the historical vehicle/negative control data (e.g. 95% control limits). The assay result was considered clearly negative when none of those criteria were met. The result was considered equivocal when neither the positive nor the negative criteria were met.

### Big Blue^®^ Rat Transgenic Gene Mutation Assay for somatic cells (liver and bone marrow) and germ cells (cauda sperm)

*In vivo* transgenic mutation assays at the *cII* locus were conducted after administration of molnupiravir or vehicle (methylcellulose, 1% [w/v], in deionized water) at a dose volume of 10 ml/kg once daily by oral gavage to at least 5 Big Blue^®^ transgenic F344 male rats (Taconic Biosciences, Inc., Germantown, NY) at 0, 50, 150, and 500 mg/kg/d for 28 consecutive days. The high dose of 500 mg/kg/d was the maximum tolerated dose.

For somatic cells (liver and bone marrow), necropsy was performed on day 31 after treatment initiation, whereas for germ cells (cauda sperm), necropsy was performed on day 98 after treatment initiation. Animals were humanely euthanized by inhalation of carbon dioxide. At necropsy, the liver (right medial lobe), bone marrow, and cauda epididymides were collected, flash-frozen in liquid nitrogen, and stored in a freezer at −80°C.

Tissues for mutation evaluation were collected from the first 5 animals per group, and then processed for DNA isolation. The DNA isolation was different for somatic cells and germ cells. For somatic cells, *cII* mutant analysis was based on Agilent RecoverEase DNA Isolation methods (Agilent, Santa Clara, CA). DNA isolated from previously treated positive control animals (Big Blue^®^ transgenic F344 male rats exposed to N-Nitroso-N-ethylurea [ENU, CAS no. 759-73-9, lot MKCN4387] by oral gavage at 20 mg/kg/d on 6 nonconsecutive days) was used as a packaging positive control. ENU is a potent direct-acting mutagen shown to be mutagenic in the target tissues.

For germ cells, genomic DNA was isolated from the frozen cauda epididymis based on a modified phenol–chloroform method described by O’Brien et al. (2014). Isolated DNA was processed using packaging extract supplied by Agilent to isolate the recoverable lambda shuttle DNA vectors from the genomic DNA and to package the lambda shuttle vector DNA into empty phage capsids, creating infectious phage particles. The methods followed were based on the Agilent instruction manual λ Select-*cII* Mutation Detection System for Big Blue Rodents (https://www.agilent.com/cs/library/usermanuals/public/720120.pdf).

Frozen stocks of *E. coli* strain G1250 originally provided and characterized by Agilent as part of the λ Select-*cII* Mutation Detection System for Big Blue^®^ Rodents were used to prepare master bacterial plates. Several colonies were picked from master plates and used to prepare overnight suspension cultures for plating of phage for plaque formation. The following day, packaged phage was adsorbed onto *E. coli* G1250 suspension cultures for at least 30 min, molten top agar was added, and the cells were plated onto bottom agar plates. Packaged phage was incubated overnight at 37°C ± 2.0°C, then scored for plaque formation and titer determination; cII mutant selection plates were incubated for 2 d (nominally 40 to 48 h) at 24°C ± 0.5°C, then scored for mutant plaques after incubation and at least 125,000 (somatic) to 200,000 (germ cell) phages were evaluated from at least 2 packagings. Big Blue^®^ transgenic rats have multiple copies of a λ shuttle vector with a cII reporter gene integrated into the genome of each cell in the body. Mutations are detected by recovering the cII gene and analyzing the phenotype of the reporter gene in a bacterial host deficient for the reporter gene (Organisation for Economic Co-operation and Development (OECD) 2020, 2022).

Mutant frequency was calculated (number of mutant phages/number of total phages screened) for each tissue specimen analyzed from each animal. An individual animal is considered the experimental unit. The assumption was that each plaque represented 1 phage that had infected 1 *E. coli* cell. Because this ratio is extremely small and may not be normally distributed, a log_10_ transformation of the mutation frequency data was performed.

The statistical analysis of mutation frequency was conducted on log_10_-transformed mutation frequency data from the vehicle control, and treated groups were evaluated using one-way ANOVA with the Dunnett correction. Mutation frequency for each dose group is represented as the mean of all animals in the dose group.

The result of the assay was considered positive when a statistically significant (*P* ≤ 0.05) increase in the frequency of cII mutants occurred, a dose-related increase was observed (trend), and the mean mutant frequency was outside the upper 95% control limits of the historical background mean mutant frequency. Biological significance and dose-response are important considerations when determining a positive result of the assay (mutagenic). The result of the assay was considered negative when no statistically significant increase in *cII* mutant frequency was observed.

### Six-month carcinogenicity study in rasH2-Tg transgenic mice

Male and female rasH2-Tg transgenic mice (Taconic Biosciences, Inc.) were administered molnupiravir orally once daily by oral gavage at doses of 0 (control group 1), 0 (control group 2), 30, 100, or 300 mg/kg/d for approximately 6 mo (26 wk). Molnupiravir and vehicle control (methylcellulose, 1% [w/v], in deionized water) formulations were administered at a dose volume of 10 ml/kg. In addition, a group of 15 mice/sex was administered positive control test material N-Nitroso-N-ethylurea as a single intraperitoneal dose. The high dose of molnupiravir 300 mg/kg/d was established as the maximum tolerated dose, based on greater than 30% decreases in body weight gain observed at higher doses in a 28-d dose range-finding study.

The animals in the carcinogenicity study underwent a complete necropsy examination, which included evaluation of the carcass and musculoskeletal system; all external surfaces and orifices; the cranial cavity and external surfaces of the brain; and the thoracic, abdominal, and pelvic cavities with their associated organs and tissue. Tissue from an extensive list was collected from all animals and preserved in neutral buffered formalin, 10% (including masses and grossly observed lesions). The collected tissues were trimmed, embedded in paraffin, sectioned, mounted on glass slides, and stained with hematoxylin and eosin. Glass coverslips were laid on top. Histopathological evaluation and peer review were performed by board-certified veterinary pathologists. To aid in the evaluation of carcinogenetic potential, a trend analysis was performed to determine whether there was a statistically significant (*P* *≤* 0.05) effect on unscheduled death in female and male mice. In addition, the incidence of various tumor types was analyzed to determine whether a statistically significant (*P* *≤* 0.05) trend was seen, with adjustments made for potentially confounding factors (e.g. mortality, time to tumor growth).

## Results

A comprehensive set of standard genotoxicity tests and supplemental *in vivo* studies were conducted to support the genotoxicity and carcinogenicity assessment of molnupiravir.

### Assessment of chromosome damage in vitro and in vivo

**Fig. 2.**
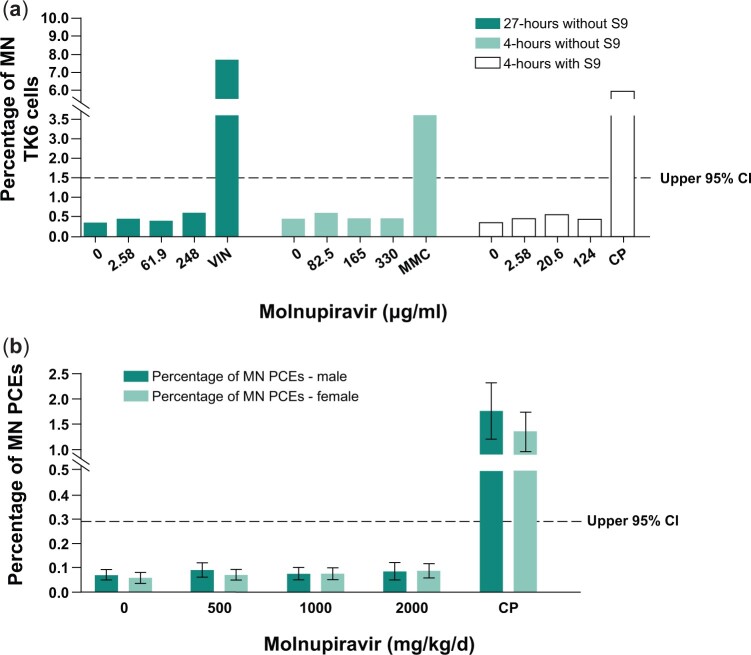
*In vitro* (a) and *in vivo* (b) micronucleus assay for chromosome breakage and chromosome loss after treatment with molnupiravir. Positive controls: CP, MMC, VIN. The dotted line represents the upper 95% CI of the historical vehicle control distribution. CI, confidence interval; CP, cyclophosphamide; MMC, mitomycin C; MN, micronucleated; PCE, polychromatic erythrocyte; S9, Aroclor 1254-induced rat liver; VIN, vinblastine.

Molnupiravir did not cause chromosome damage *in vitro* or *in vivo* when assessed in the micronucleus assay. Micronuclei can be induced by a wide range of changes in chromosome integrity, such as chromosome breaks, translocations, and whole chromosome loss. Such changes can result from direct reactivity with DNA or from inhibition of enzymes that impact chromosome integrity (International Council for Harmonisation (ICH) Harmonised Tripartite Guideline 2011). Molnupiravir was negative in the *in vitro* assay at all tested concentrations, ranging from 0.650 to 330 μg/ml (∼1 mM) (Fig. 2a). The highest concentration assessed in the 27-h continuous treatment and the 4-h treatment with S9 were limited by toxicity and exhibited 58% and 56% toxicity, respectively. The 4-h treatment without S9 was tested up to 1 mM, the highest concentration recommended by ICH S2R1 guideline. The lack of chromosome damage was confirmed in an *in vivo* study in which molnupiravir did not cause an increase in the number of micronuclei in PCEs in rat bone marrow following 2 consecutive days of oral treatment up to 2,000 mg/kg/d (Fig. 2b). Because molnupiravir is rapidly converted to NHC in both *in vitro* and *in vivo* test systems (Hernandez-Santiago et al. 2004), exposure to molnupiravir is considered indicative of exposure to NHC as well; data demonstrate NHC exposure in Sprague–Dawley rats (Table S1).

### Assessment of mutagenicity in vitro

Molnupiravir and NHC were independently evaluated for their potential to induce mutations in the bacterial reverse mutation assay (Ames) assay. The Ames assay measures small base pair changes (mutations) in the genome. A positive response in this assay can occur through direct reactivity with DNA, intercalation between base pairs, oxidative stress, and impairment of DNA repair enzymes, and can sometimes be a result of bacteria-specific effects of a compound. A set of standard bacterial strains is used in the assay, each containing mutations of specific loci such that a reverse mutation event leads to growth on restrictive media and is conducted with and without an exogenous S9 metabolic activation system. Strain specificity for mutation induction can provide insight into the mechanism of mutagenicity, and a positive response in any one strain leads to a positive result in the assay.

**Fig. 3.**
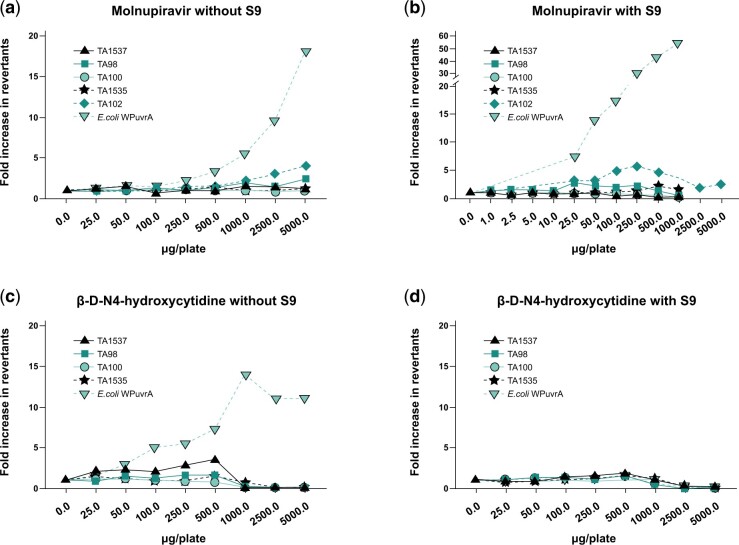
Molnupiravir (a/b) and β-D-N4-hydroxycytidine (c/d) Ames assay results without (a and c) and with (b and d) S9 metabolic activation. All strains were assessed up to either 5,000 µg/plate or toxicity-limited concentrations. S9, Aroclor 1254-induced rat liver.

Treatment with molnupiravir induced increases in the number of revertant colonies at ≥25.0 μg/plate in TA102 and WP2*uvr*A strains with metabolic activation and at ≥500 μg/plate in the WP2uvrA strain and ≥1,000 μg/plate in the TA102 strain without metabolic activation (S9). Treatment with NHC induced increases in mutation frequency starting at approximately the same concentration as molnupiravir in theWP2*uvr*A strain. NHC was not assessed in *S. typhimurium* TA102 (Fig. 3).

As a follow-up to the *in vitro* results, an assessment of *in vivo* mutagenicity was completed.

### Assessment of mutagenicity in vivo

#### *In vivo* erythrocyte Pig-a gene mutation assay

**Fig. 4.**
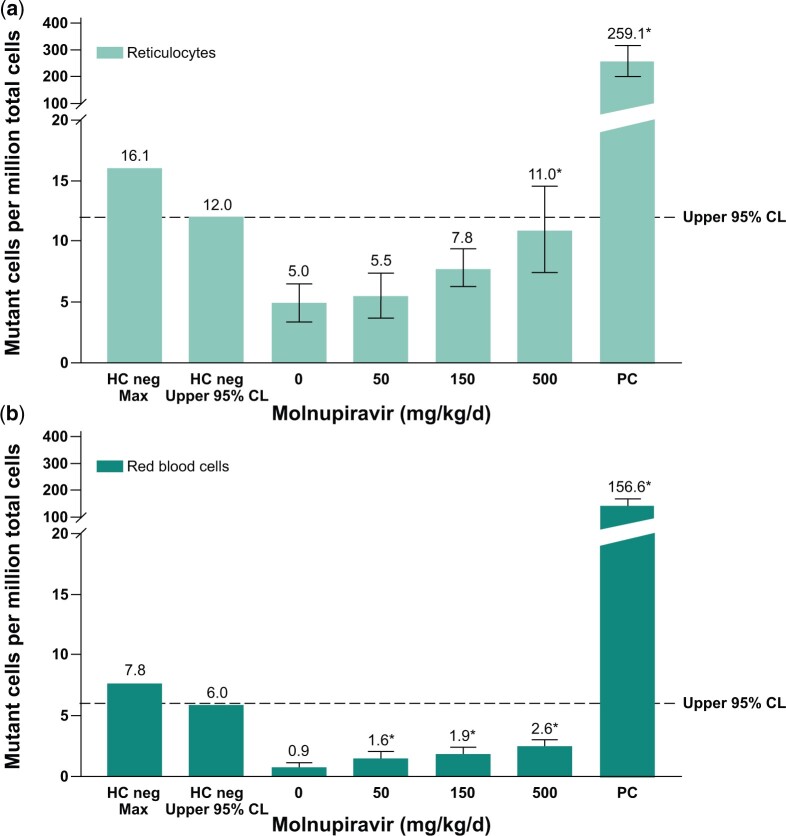
Pig-a mutation assay in rats after 28-d treatment with molnupiravir. Mutation frequency in reticulocytes (a) and mutation frequency in red blood cells (b). Positive control: ENU 20 mg/kg administered on 6 nonconsecutive days; *P ≤ 0.05. Dotted line represents upper 95% CL of the historical vehicle control study mean distribution. CL, confidence limit; ENU, N-Nitroso-N-ethylurea; HC, historical control; PC, positive control.

Mutations in the endogenous X-linked Pig-a gene were evaluated in RETs and RBCs that originated from rapidly proliferating erythroid precursor cells. Data are summarized in Fig. 4. Mean RBC and RET mutation rates for all treated groups were within the range of the 95% upper limit of historical negative control values observed in the laboratory; however, slight but statistically significant increases (*P* ≤ 0.05) in the incidence of mutant RBCs were observed at all dose levels, as well as of mutant RETs at 500 mg/kg/d compared with the concurrent negative control. The increases did not demonstrate a statistically significant dose-related trend when evaluated using the Cochran–Armitage trend test. Additionally, the concurrent negative control values in this assay were variable and on the low end of the historical control of the laboratory. In light of the data, the assay results were deemed equivocal, aligned with recommendations by the International Workshop on Genotoxicity Testing (IWGT) Workgroup for the *in vivo* Pig-a Assay (Gollapudi et al. 2015). The positive control treatment ENU (20 mg/kg) produced a statistically significant (*P *<* *0.05) and robust increase in mutant frequencies in RBCs and RETs. At the time, this study was conducted, the Pig-a Assay OECD guideline describing laboratory training and proficiency investigations was not available. Therefore, a follow-up assessment of *in vivo* mutation potential was performed using the OECD-compliant Big Blue^®^ Transgenic Rodent Gene Mutation Assay.

### Big Blue^®^ Transgenic Rat Gene Mutation Assay in somatic cells (liver and bone marrow) and germ cells (cauda sperm)

A transgenic rodent mutation (TGR) assay, such as the Big Blue^®^ TGR Assay in F344 transgenic rats, is considered the gold standard for *in vivo* assessment of a compound’s mutagenic potential and as a follow-up to *in vitro* positive mutagenicity findings (Eastmond et al. 2009; International Council for Harmonisation (ICH) Harmonised Tripartite Guideline 2011; International Council for Harmonisation (ICH) Harmonised Tripartite Guideline 2017; Robison et al. 2021). The Big Blue^®^ TGR Assay can detect minimal genetic damage that may be caused by mispairing or misincorporation of bases during replication (Lambert et al. 2005).

**Fig. 5.**
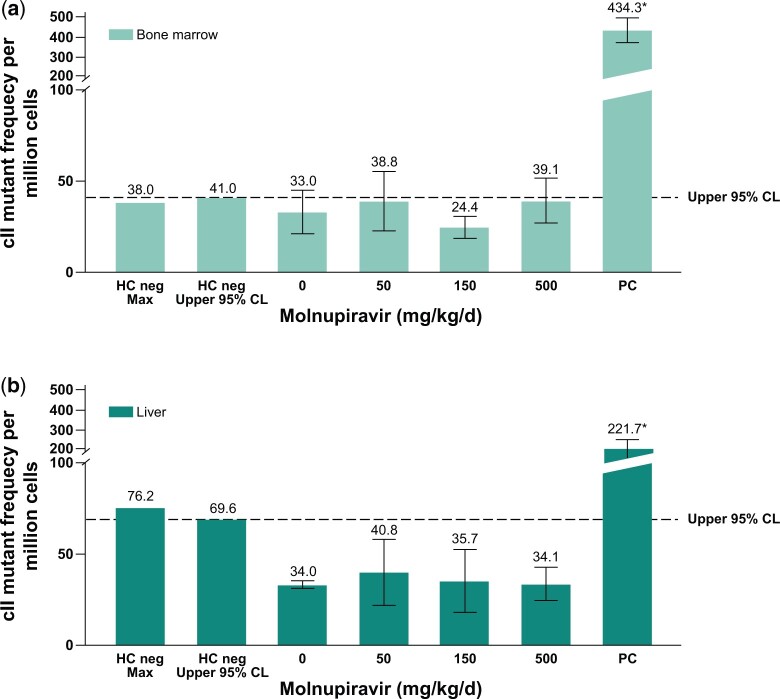
Big Blue^®^ Transgenic Rat Gene Mutation Assay in somatic cells after 28-d treatment with molnupiravir. Mutation frequency in bone marrow (a) and mutation frequency in liver cells (b). The dotted line represents the upper 95% CL of the historical vehicle control study mean distribution. Positive control: ENU 20 mg/kg administered on 6 nonconsecutive days (ENU); *P ≤ 0.05. CL, confidence limit; ENU, N-Nitroso-N-ethylurea; HC, historical control; PC, positive control.

Bone marrow is a rapidly proliferating tissue and is particularly useful as a follow-up to the equivocal signal observed in RETs and RBC (derived from bone marrow) in the Pig-a assay. The liver is a more slowly proliferating tissue that was chosen based on its high metabolic activity and exposure to all absorbed drug during the first pass (>90% of the dose is absorbed in rats). Molnupiravir was tested using a Big Blue^®^ TGR Assay and somatic mutations were analyzed in the bone marrow and liver. Molnupiravir did not cause a statistically significant increase in mean mutant frequency at the cII gene in the liver or the bone marrow of Big Blue^®^ rats at any dose level compared with concurrent vehicle control, and the mean mutant frequency in the molnupiravir-treated groups did not exceed the historical control 95% upper limit (Fig. 5). Additionally, none of the individual animal mutation frequencies were outside the 95% upper control limit for individual animals (bone marrow individual upper limit = 58.4 × 10^−6^ and liver individual upper limit = 86.0 × 10^−6^). The laboratory historical vehicle control mean mutant frequency for the bone marrow was 30.0 ± 14.2 × 10^−6^ (*n *=* *55) and for the liver was 44.0 ± 21.0 × 10^−6^ (*n *=* *109). The positive control, ENU (20 mg/kg), produced a statistically significant (*P* ≤ 0.05) and robust increase in mutant frequencies in both the liver and the bone marrow, consistent with the laboratory positive control mean frequency of 408.3 ± 177.5 × 10^−6^ (bone marrow) and 216.1 ± 110.1 × 10^−6^ (liver).

Analysis of NHC-TP exposure in tissue specimens across multiple rat studies indicated that the peak concentrations in bone marrow and liver are generally consistent with the other major tissue types sampled, having either similar or greater mean values (Fig. S1 and Table S1). NHC and NHC-TP have a wide tissue distribution given the ubiquitous expression of nucleoside uptake transporters (Miller et al. 2021) and the similarity of NHC to endogenous cytidine (1 added hydroxyl group). Additionally, the cellular housekeeping enzymes responsible for nucleoside/nucleotide metabolism are expressed and regulated in all cell types (Lane and Fan 2015); therefore, broad intracellular exposure to NHC-TP and other nucleoside-derived metabolites would be expected as a result of metabolism in mammals.

**Fig. 6.**
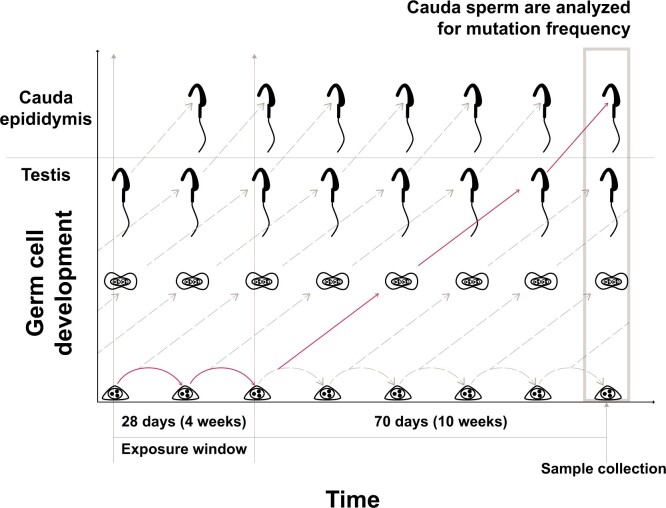
Germ cell development over a 28-d exposure window and 70-d treatment-free periods accounts for the spermatogenic cycle kinetics in rats and maximizes exposure in undifferentiated spermatogonia (stem cells). The full line denotes the developmental trajectory of spermatogonia that received a full 28-d exposure to molnupiravir into sperm that were collected from the cauda epididymis at the end of the treatment-free period.

In addition, molnupiravir was tested in Big Blue^®^ TGR Assay gem cells (cauda epididymis) after 28 d of treatment followed by a 70-d treatment-free period. The revised OECD Test Guideline 488 (Organisation for Economic Co-operation and Development (OECD) 2020, 2022) outlines the measurement of mutations in germ cells collected from seminiferous tubules after 28-d dosing with a 28-d treatment-free period. To study the potential of molnupiravir to induce germ cell mutations in sperm from the cauda epididymis of rats, an optimized study design for the detection of permanent mutations in spermatogonial stem cells was used. In order to achieve optimized sensitivity in stem cells and given the timing of the rat spermatogenesis cycle (Fig. 6), the sampling time for caudal rat sperm was 70 d after the end of the 28-d administration period as per OECD TG488. Laboratory proficiency for the optimized protocol (28-d treatment and 70-d treatment free) was demonstrated and a historical control database for mutation frequency of vehicle and positive controls was established before initiating the study of molnupiravir. The robustness of the cauda epididymis sperm mutation method and interstudy variability was tested in 4 independent experiments, and the data were used to populate a historical control database. In these experiments, animals were administered ENU 20 mg/kg per dose by oral gavage as the positive control on study days 1, 2, 3, 12, 19, and 26, and the negative control group included animals treated with vehicle (methylcellulose, 1.0% [w/v], in deionized water) daily for 28 d. The 4 independent experiments yield negative control mutant frequencies of 47.9 ± 19.8 × 10^−6^ (*n *=* *47) and positive control mutant frequencies of 206.4 ± 38.5 × 10^−6^ (*n *=* *31), thus establishing the responsiveness of the model system and consistency of methods in accordance with OECD Test Guideline 488 for establishing a historical control database and laboratory proficiency (Organisation for Economic Co-operation and Development (OECD) 2020, 2022).

**Fig. 7.**
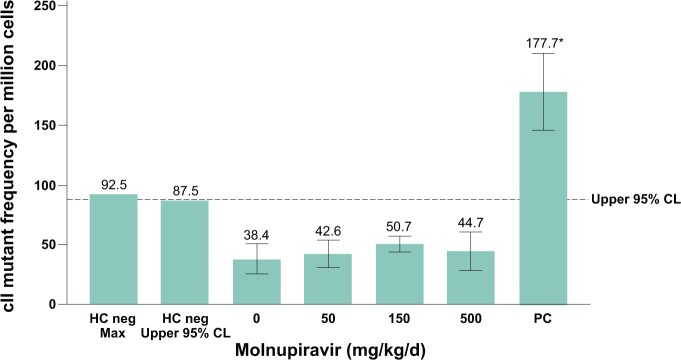
Big Blue^®^ Transgenic Rat Gene Mutation Assay in sperm from cauda epididymis after 28-d treatment and a 70-d treatment-free period. The vehicle was methylcellulose, 1% (wt/vol), in deionized water. The dotted line represents the upper 95% CL of the historical vehicle control study mean distribution. Positive control: ENU dose was 20 mg/kg administered on days 1, 2, 3, 12, 19, and 26. CL, confidence limit; ENU, N-Nitroso-N-ethylurea; HC, historical control; PC, historical positive control.

Molnupiravir did not cause a statistically significant increase in mutant frequency at the cII gene in sperm from the cauda epididymis at any dose tested compared with the concurrent vehicle control (Fig. 7). The mean of all molnupiravir-treated groups and individual animal mutant frequencies were within the 95% control limits of historical negative control data for mutant frequency in sperm from the cauda epididymis, and there was no evidence of a dose-dependent increase in mutations. In a separate study, NHC and NHC-TP levels were measured in the testis of wild-type F344 rats after 14 d of daily treatment with molnupiravir 500 and 750 mg/kg/d (Supplementary Material). Measurable levels of both catabolites were detected, demonstrating exposure in the tissue assessed for mutagenicity.

### Carcinogenicity assessment in a 6-mo rasH2-Tg mouse study

A 6-mo carcinogenicity study in rasH2-Tg mice was performed to assess the carcinogenic potential of molnupiravir. The CByB6F1/Tg(rasH2)2Jic hemizygous mouse (rasH2-Tg mouse) was selected as the animal model for carcinogenicity assessment based on its regulatory acceptance in accordance with ICH S1B(R1). The rasH2-Tg mouse carries multiple copies of a human Ha-ras oncogene and is used in short-term carcinogenicity models because the rasH2-Tg mouse develops neoplasms earlier than conventional mice and rats and has been shown to be sensitive at detecting both genotoxic and nongenotoxic carcinogens (Morton et al. 2002; Hisada et al. 2022). In a recently published comprehensive survey of 53 pharmaceuticals that have been tested in a 6-mo rasH2-Tg mouse study, this model was shown to be similarly sensitive to the 2-yr rat and 2-yr mouse studies for genotoxic carcinogens (Hisada et al. 2022).

Administration of molnupiravir 0, 30, 100, and 300 mg/kg/d once daily by oral gavage for approximately 26 wk to rasH2-Tg mice did not result in any carcinogenic effects at any dose level. The high dose of 300 mg/kg/d was established as the maximum tolerated dose. There were no molnupiravir-related effects on mortality, clinical observations, body weight or body weight gain, food consumption, and macroscopic or microscopic findings at any dose level. NHC plasma concentrations (0.5 h post dose on day 182) were 7.4, 18.7, and 60.1 μM at 30, 100, and 300 mg/kg, representing approximately 0.7-, 1.9-, and 6-fold the clinical *C*_max_ (10 μM), respectively. Although the positive control group (N-Nitroso-N-methylurea, a DNA-alkylating agent) exhibited the expected neoplastic changes (e.g. increased incidence of lymphoma and papilloma in various tissue types), there were no molnupiravir-related neoplastic or nonneoplastic findings at any dose level across a robustly characterized dose range up to the maximum tolerated dose. Tumor incidence data and statistical values are provided in Table 1.

**Table 1.** Summary incidence of primary neoplasms: molnupiravir 26-wk oral gavage carcinogenicity study in rasH2-Tg mice.

Dose (mg/kg)	0	0	30	100	300	75
	Control 1	Control 2	MOV	MOV	MOV	NMU
	M/F	M/F	M/F	M/F	M/F	M/F
N	25/25	25/25	25/25	25/25	25/25	15/15
Total neoplasms	8/1	6/3	2/4	3/4	4/6	34/39
Adenoma						
Lung (bronchoalveolar)	2/0	1/0	0/0	1/0	0/1	3/0
Harderian gland	1/0	0/0	1/0	0/0	0/0	2/1
Carcinoma						
Lung (bronchoalveolar)	2/0	1/1	0/0	1/1	2/1	0/1
Other	0/0	0/0	0/0	0/0	0/0	1/3
Hemangiosarcoma^a^						
Spleen	1/0	1/0	1/2	1/0	1/3*	4*/0
Other	2/1	2/2	0/2	0/1	0/1	1/1
Lymphoma (primary site undetermined)	0/0	0/0	0/0	0/0	1/0	5***/7***
Polyp						
Uterus/cervix	–/0	–/0	–/0	–/0	–/0	–/2*
Papilloma	0/0		0/0		0/0	
Esophagus						2*/3**
Stomach		1/0				10***/9***
Skin				0/2		6***/7***
Urethra						0/2*
Urinary bladder						0/2**
Vagina						0/2*

a A statistically significant (*P *=* *0.032; multiplicity adjusted *P *=* *0.040) increasing trend in tumor incidence through 300 mg/kg/d was observed for hemangiosarcoma in the spleen (12%) in female mice. Step-down analysis results through 100 mg/kg/d were not statistically significant (*P *=* *0.470). The marginally significant *P*-value through 300 mg/kg/d was attributed to the random occurrence of an unusually low concurrent control rate (0%) (U.S. Food and Drug Administration (FDA) Center for Drug Evaluation and Research (CDER). Guidance for Industry 2001) and was not test article related given that the incidence was well within the historical control range for female CByB6F1/Tg rasH2 mice from the test facility (0% to 16%) and in the published literature (0% to 16% Paranjpe et al. 2013, 2019; 0% to 26% Nambiar et al. 2012).

MOV, molnupiravir; NMU, N-Nitroso-N-methylurea; M, male; F, female.

*** *P* ≤ 0.001,

** *P* ≤ 0.01,

* *P* ≤ 0.05 (one-sided *P*-value from upper-tail Fisher exact test).

## Discussion

The genotoxic and carcinogenic potential of molnupiravir have been comprehensively tested. The weight of evidence supports the lack of chromosomal damage or mutagenicity in somatic and germ cells when molnupiravir was tested in multiple *in vivo* mutation studies at doses approximately 19-fold higher than the clinical dose (400 mg every 12 h, or approximately 27 mg/kg/d for a 60-kg individual) and administered for a duration 5 times longer than the clinical regimen of 5 d. Additionally, no evidence of carcinogenicity was observed at doses approximately 11-fold higher than the clinical dose and administered for approximately 36 times longer than the clinical regimen. Dose or exposure multiples based on body surface area or NHC plasma exposures in the rodent mutagenicity and carcinogenicity studies were also equal to or exceeding those observed clinically at the recommended human dose of molnupiravir. Although molnupiravir appears to induce mutations under *in vitro* conditions in bacteria and immortalized cell lines, molnupiravir did not induce mutations *in vivo*.

In the *in vitro* bacterial reverse mutation assay, exposure of bacterial cells to molnupiravir or NHC induced mutations only in *S. typhimurium* TA102 and *E. coli WP2 uvrA* strains. The target for mutation in these strains is the unique AT sequence. Reversion to the wild-type function of the selection gene requires a transition mutation that changes an A to a G. This type of mutation is analogous to those observed in viral RNA with the incorporation of NHC by the viral RNA-dependent RNA polymerase, leading to base pairing with either G or A and resulting in an increased frequency of transition mutations (e.g. G to A or A to G) (Kabinger et al. 2021). The *S. typhimurium* TA102 and *E. coli WP2 uvrA* strains are also known to detect oxidative DNA lesions. In addition, a cellular response to oxidative stress was observed after treating mammalian cells with molnupiravir *in vitro* in 2 independent studies (Brandsma et al. 2022; Kobayashi et al. 2023). Thus, oxidative stress could potentially be one of the mechanisms of mutation induction *in vitro*.

In mammalian cells, mutagenicity was reported in 2 independent *in vitro* studies (Zhou et al. 2021; Miranda et al. 2022). Zhou et al. (2021) conducted nonconventional hypoxanthine-guanine phosphoribosyltransferase assays in Chinese hamster ovary cells, in which cells treated with NHC continuously for 32 d showed a concentration-dependent increase in mutations. Of note, the conditions used for this assay were significantly different from the conditions of standard protocols and OECD guidelines (Troth et al. 2021). Using a novel HiFi sequencing technique, Miranda et al. (2022) showed that mutations increased in mouse lymphoma L5178Y cells and human lymphoblastoid TK6 cells after 5 d of continuous treatment with molnupiravir and NHC. Furthermore, the whole-genome HiFi sequencing analysis showed statistically significant induction of mutations, with the majority of molnupiravir- and NHC-induced mutations being A:T to G:C transitions, consistent with previously reported results in bacteria. Potential reasons for the *in vitro* mutations may be attributed to either the incorporation of the ribonucleotide triphosphate of NHC by the mammalian/bacteria DNA polymerase or the formation of a theoretical deoxy-NHC-triphosphate metabolite, which is subsequently incorporated into DNA. Considering that deoxy-NHC-triphosphate has not been observed in rat and human *in vitro* hepatocyte assays or plasma and urine samples *in vivo* (using authentic standard and high-resolution liquid chromatography-mass spectrometry/mass spectrometry) and that ribonucleotides are efficiently excluded and removed from DNA during synthesis, other factors likely contribute to the *in vitro* positive results (Hernandez-Santiago et al. 2004; Williams et al. 2016). Among the factors to be considered is the impact of static cell culture conditions resulting in high intracellular accumulation of NHC, NHC-TP, and other potential anabolites/catabolites and the extensive formation of the natural pyrimidines C and U. The persistent static and high *in vitro* concentrations of NHC, unlikely to occur in an *in vivo* mammalian system with excretion capacity, could result in confounding *in vitro* outcomes based on artificially higher intracellular accumulation of phosphorylated NHC nucleotides (monophosphate, diphosphate, and triphosphate), which are cell membrane impermeable and, therefore, trapped intracellularly. In an *in vitro* cell culture, the continuous exposure to an abundant supply of NHC may result in nonphysiologically meaningful intracellular concentrations of NHC-derived nucleotides over time. *In vitro* cell culture conditions do not replicate the dynamic conditions of an *in vivo* test system, particularly with a nucleoside analog that is also rapidly metabolized to natural nucleosides *in vivo*. Other metabolic differences of immortalized cell lines leading to NHC *in vitro* mutagenicity are transcriptional differences of enzymes associated with pyrimidine metabolism (Xu et al. 2023).

Interestingly, a similar lack of translation of *in vitro* to *in vivo* results was reported in studies comparing mammalian host cell RNA mutations following molnupiravir treatment. In cell culture experiments using human bronchiolar and tracheal epithelial cells, NHC concentrations between 10 and 50 μM induced detectable and dose-related C to U transition mutations in host mRNA (Sheahan et al. 2020). However, no increases in the number of C to U transitions were observed in bronchial epithelial cell mRNA collected from mice given up to 500 mg/kg molnupiravir twice daily, corresponding to maximum drug concentrations (>100 μM) greatly exceeding the concentrations at which transition mutations of mRNA were observed *in vitro* (Sheahan et al. 2020). A similar *in vivo* efficacy study conducted in ferrets demonstrated no increase in C to U transitions in tumor necrosis factor α or cyclooxygenase-15 mRNA after molnupiravir treatment at 100 mg/kg every 12 h (Toots et al. 2019). In each of these studies, robust and dose-related C to U transition signatures were observed in the viral genome following treatment but not in the host mRNA. Considering that very high plasma concentrations of NHC had no detectable effect on RNA transitions *in vivo*, the potential for *in vivo* DNA effects, either via ribonucleoside (mis)incorporation or deoxynucleoside formation, seems unlikely.

Regulatory guidelines and expert opinion recognize that *in vitro* systems do not always accurately replicate *in vivo* cellular activities and extracellular factors (e.g. metabolism and excretion) and recommend *in vivo* assessment as the appropriate follow-up to a potential hazard identified *in vitro* (Eastmond et al. 2009; International Council for Harmonisation (ICH) Harmonised Tripartite Guideline 2011; International Council for Harmonisation (ICH) Harmonised Tripartite Guideline 2017; Robison et al. 2021). Consistent with scientific expectations, follow-up to an *in vitro* mutation signal was completed for molnupiravir. Specifically, molnupiravir was assessed in 2 distinct rodent mutagenicity models: The Pig-a mutagenicity assay and the Big Blue^®^ (cII locus) Transgenic Rodent Gene Mutation Assay in somatic and germ cells. In the Pig-a assay, the results were inconclusive, and the study was deemed equivocal. Molnupiravir was clearly negative in the Big Blue^®^ Transgenic Rodent Gene Mutation Assay in somatic cells collected from the liver and bone marrow, and molnupiravir was also negative in male germ cells.

The Big Blue^®^ Transgenic Rodent Gene Mutation Assay in F344 rats has sensitively detected human carcinogens (Lambert et al. 2005), including nucleoside analogs such as azathioprine (Smith et al. 1999) and 5-(2-chloroethyl)-2ʹ-deoxyuridine (Suter et al. 2004). Zeller et al. (2018) reported high sensitivity in detecting human carcinogens using this assay either alone (90%) or combined with micronucleus data (93%). Similar results were described in a review showing 92% positive predictivity of a transgenic rodent gene mutation assay for 154 rodent carcinogens (Lambert et al. 2005). Since the ultimate goal of conducting genotoxicity assessments is to detect carcinogenic potential, the lack of carcinogenicity when molnupiravir was administered for 26 wk to rasH2-Tg mice further strengthens the evidence that transgenic rodent gene mutation assays are highly predictive of carcinogenic outcomes. Additionally, lack of mutagenicity in somatic cells has been predictive of lack of mutagenicity in germ cells, and no unique germ cell mutagens have been identified (Holden 1982; Waters et al. 1994; Eastmond et al. 2009; Yauk et al. 2015). Consistent with lack of mutagenicity and carcinogenicity in somatic cells, the results from the *in vivo* germ cell mutation assay in Big Blue^®^ transgenic male rats indicate that molnupiravir is not mutagenic in germ cells.

NHC is rapidly metabolized *in vivo* and requires biotransformation for activity. A radiolabeled study in rats demonstrated that molnupiravir-related radioactivity was completely absorbed (>90%) and the bioavailability of NHC after administration of molnupiravir was high (>50%) (data not shown). Because NHC exposure in rat plasma and tissues increased linearly with the dose, near-complete absorption must be assumed at all doses in the mutagenicity studies. Therefore, the amount of NHC metabolites/anabolites formed in these *in vivo* studies is directly correlated with the dose. Considering that the *in vivo* mutation studies were conducted at doses up to 19 times the clinical dose, the amount of anabolites/catabolites of NHC formed and tested in these assays is markedly higher than the amount that could be formed at much lower clinical doses. Therefore, if there is any potential for NHC metabolism to form deoxy-NHC-triphosphate in vivo, that potential was tested in the in vivo mutation studies and carcinogenicity study at a much higher body burden of NHC anabolites/catabolites and over a longer period of molnupiravir administration than the clinical regimen.

In summary, molnupiravir was comprehensively assessed for genotoxicity and carcinogenicity potential, in accordance with expert opinion and international guidelines (multi-agency alignment in ICH, OECD, and expert international bodies). These *in vivo* studies were conducted to assess mutagenicity in Big Blue^®^ rats (at the cII locus) in liver, bone marrow, or sperm; in Sprague–Dawley rats (at the Pig-a locus) in RBCs; and carcinogenicity in rasH2-Tg mice support the conclusion that the data do not indicate a risk of genotoxicity in somatic or germ cells or carcinogenicity for patients taking molnupiravir.

## Supplementary Material

kfae112_Supplementary_Data
