# Supplementary material for: Comprehensive genotoxicity and carcinogenicity assessment of molnupiravir
Source: Toxicol Sci. 2024 Sep 20;202(2):278–90. doi: 10.1093/toxsci/kfae112 (PMC11589102; doi:10.1093/toxsci/kfae112)
Supplement: kfae112_Supplementary_Data [file kfae112_supplementary_data.docx]

**Supplementary Material**

**Comprehensive Genotoxicity and Carcinogenicity Assessment of Molnupiravir**

Patricia A. Escobar*, Zhanna Sobol, Randy R. Miller, Sandrine Ferry-Martin, Angela Stermer1, Binod Jacob, Nagaraja Muniappa, Rosa Sanchez, Alema Galijatovic-Idrizbegovic, Rupesh P. Amin, Sean P. Troth

Nonclinical Drug Safety and Pharmacokinetics Dynamics Metabolism and Bioanalysis, Preclinical Development, Merck & Co. Inc., Rahway, New Jersey, USA

**Supplementary Figure 1. Dose-standardized tissue distribution of NHC-TP in rats treated with molnupiravir.**

**
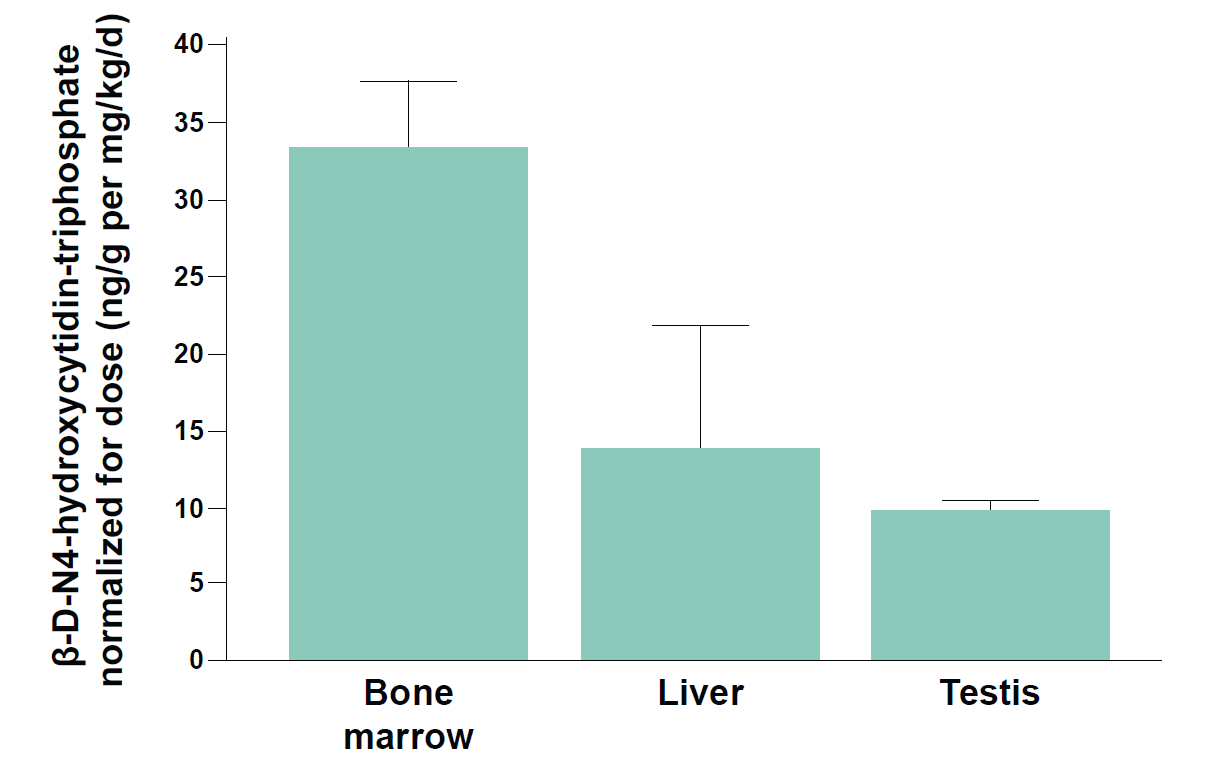
**

Tissue distribution of NHC-TP after oral treatment with molnupiravir. Data shown are from independent studies conducted with male rats, and samples were collected at approximately the C_max_ for NHC-TP (1–3 h after dose). Bone marrow samples were collected after a single oral treatment with molnupiravir 50, 150, and 500 mg/kg/d. Liver samples were collected from 2 independent studies after a single oral treatment with molnupiravir 200, 320, and 700 mg/kg. Testis samples were collected after 14 days of daily treatment with molnupiravir 500 and 750 mg/kg/d. The data shown represent the dose-standardized average. Details and data for additional tissue samples can be found in Supplemental Table 1.

C_max_, maximum drug concentration; NHC-TP, β-D-N4-hydroxycytidine-triphosphate.

**Supplementary Table 1. NHC-TP tissue exposure concentration data collected at approximately T_max_ on day 1 only in male rats treated with molnupiravir**

| **Tissue** | **Molnupiravir dose (mg/kg/d)** | **NHC-TP tissue concentration (µg/g)** | **Standard deviation** | **Dose-standardized NHC-TP tissue concentration (ng/g) per molnupiravir dose (mg/kg/d)** |
| --- | --- | --- | --- | --- |
| Bone marrow | 50 | 1.9 | 0.4 | 38.4 |
|  | 150 | 4.7 | 1.2 | 31.4 |
|  | 500 | 15.1 | 4.9 | 30.2 |
|  |  |  |  |  |
| Brain | 200 | 1.7 | 0.0 | 8.5 |
|  | 700 | 6.1 | 0.5 | 8.8 |
|  |  |  |  |  |
| Heart | 200 | 2.7 | 0.2 | 13.7 |
|  | 320^a^ | 4.4 | 1.0 | 13.8 |
|  | 700 | 8.2 | 4.2 | 11.7 |
|  |  |  |  |  |
| Kidney | 200 | 4.5 | 1.0 | 14.1 |
|  | 320^a^ | 3.6 | 3.1 | 18.0 |
|  | 700 | 15.8 | 7.4 | 22.5 |
|  |  |  |  |  |
| Liver | 200 | 3.3 | 0.7 | 16.7 |
|  | 320^a^ | 1.5 | 1.4 | 4.7 |
|  | 700 | 14.0 | 8.3 | 20.0 |
|  |  |  |  |  |
| Lung | 200 | 1.8 | 0.5 | 9.0 |
|  | 320^a^ | 19.5 | 8.6 | 60.9 |
|  | 700 | 6.0 | 1.6 | 8.6 |
|  |  |  |  |  |
| Spleen | 200 | 4.4 | 0.2 | 22.0 |
|  | 320^a^ | 18.8 | 2.7 | 58.8 |
|  | 700 | 14.4 | 2.8 | 20.6 |
|  |  |  |  |  |
| Testis | 500 | 5.1 | 0.5 | 10.2 |
|  | 750 | 6.9 | 0.7 | 9.3 |

Aggregate data from multiple studies generated using HPLC separation followed by mass spectroscopy. Data collected in 3–8 animals. ^a^Data from 1 study. HPLC, high-performance liquid chromatography; NHC-TP, β-D-N4-hydroxycytidine-triphosphate; T_max_ time to peak drug concentration.
